# A compact wideband antenna with high gain based on spoof surface plasmon polaritons

**DOI:** 10.1038/s41598-024-54682-w

**Published:** 2024-05-02

**Authors:** Farshad Arghandeh, Bijan Abbasi-Arand, Maryam Hesari-Shermeh

**Affiliations:** Department of Electrical and Computer Engineering, Tarbiat Modares University, Tehran, 14115-194 Iran

**Keywords:** Wideband, End-fire, Spoof surface plasmon polaritons (SSPP), Electrical and electronic engineering, Metamaterials

## Abstract

In this paper, a novel wideband antenna with a simple structure and low profile based on spoof surface plasmon polaritons (SSPPs) is proposed. The structure consists of periodically modulated corrugated metal strips as transmission lines, a CPW feed, and a ground metal plate as an antenna reflector. The SSPP transmission line is used to convert quasi-TEM to SSPP mode and achieve optimal impedance matching. The prototype of the end-fire antenna has been designed and fabricated. The simulation results show that this antenna can achieve a gain of 10.19 dB, a bandwidth of 146%, and an efficiency of 90% in a wide operating band from 7 to 45 GHz. The proposed design illustrates great potential that includes high efficiency, good directivity, high gain, wide bandwidth, and easy manufacturing.

## Introduction

Surface plasmon polaritons (SPP) are light waves that can be propagated at the interface between a metal and a dielectric^1–3^. Since the metals in microwave frequencies behave as perfect electrical conductors (PEC) with negative permittivity, SPPs are not produced in these frequencies^4,5^. However, to take advantage of the properties of SPP modes at microwave frequencies, metamaterials are presented to achieve novel surface responses that have similar properties to SPP modes. In 2004, Pendry et al.^6^ designed an array structure consisting of periodic subwavelength cubic holes on a metal surface, which is referred to as a spoof structure. A spoof surface plasmon polariton (SSPP) has been fabricated using plasmonic metamaterials with alternating sheet metal structures, which exhibit SPP's behavior at microwave and terahertz frequencies^7^.

One of the key advantages of this metamaterial is that the geometrical parameters of the elements can control the dispersion and spatial confinement characteristics of SSPPs. These advantages make SSPPs good candidates for high-density integrated circuits and components at millimeter-wave and terahertz frequencies^8,9^. Significant advancements have been achieved in this area, including the development of transmission structures utilizing coplanar waveguide (CPW) or microstrip lines^10–12^, advancements in filter technology^8,13^, the design of power dividers^14^, and innovation in antennas^15–23^. In^16^, an end-fire antenna was proposed, utilizing a structure based on SSPPs with metamaterial particles. In this design, the main radiator is a printed dipole, while the SSPP waveguide serves as a feed structure for the printed dipole. The configuration includes an I-shaped resonator array to achieve a high effective refractive index, resulting in enhanced gain in the desired end-fire direction. Moreover, in^17^, a near-end fire wideband antenna was introduced, utilizing SSPPs with metamaterial H-shaped cells, while in^18^, an end-fire antenna utilizing SSPPs was proposed. To achieve the excitation of the printed dipole, a pair of SSPP waveguides was printed on both the upper and lower layers of the substrate. In^19^, a wideband end-fire antenna with a fishbone shape was designed, utilizing SSPPs. The feeding structure of that antenna comprised a microstrip-to-slit converter and a differential mode exciter, enabling the excitation of the odd mode signal on the SSPPs. In^20^, a small aperture end-fire antenna was designed using odd-mode SSPPs, and the SSPP antenna was constructed using dipole unit cells. The antenna presented in^21^ employed a printed dipole as the primary radiator while, additionally, shape resonators and parasitic bands were incorporated in the direction of the end-fire to enhance directionality. However, due to the inherent resonant characteristics of the dipole, the bandwidth of that end-fire antenna was limited, resulting in a lower gain and front-to-back ratio. Furthermore, in^22^, a near-end fire antenna based on SSPPs was presented, where the objective of that paper was to design a high-gain antenna within the frequency range of 7.5 to 8.5 GHz. In^23^, a high-gain SSPP-based broadband antenna is proposed, in which the SSPP waveguide is composed of a very thin corrugated metal strip and a dielectric substrate layer. To improve the SSPP antenna directivity, a director is also loaded at the end of the antenna. The proposed antenna has a bandwidth of 65% and a maximum gain of 6.6 dBi.

In this paper, a novel wideband antenna is introduced using an SSPP structure. In this design, a wide impedance bandwidth ranging from 7 to 45 GHz is achieved using a semi-circular unit cell, two metal plates as the antenna ground, a CPW feed, and an SSPP transmission line. The semi-circular slits are designed to match the characteristic impedance of the CPW to the SSPP transmission line and then reduce the reflection. Moreover, the proposed unit cell provides the conditions for creating a slow wave and reduced reflection, which leads to better impedance matching. By utilizing the SSPP transmission line, the antenna shows better field confinement and higher efficiency. The proposed antenna has been designed, simulated, and fabricated, and the results show that the novel end-fire antenna has the advantages of a wide impedance bandwidth (146%), high efficiency (90%), high gain (10.19 dB), and a compact size.

## Design implications

**Figure 1:**
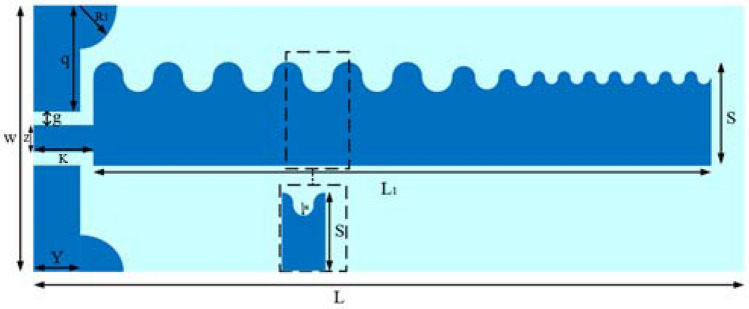
Schematic of our proposed antenna, based on SSPP.

The configuration of the proposed antenna is illustrated in Fig. 1. This antenna has been designed on a Rogers RT5880 substrate with ${\varepsilon }_{r}=2.2$ and ${\text{tan}}\delta =0.0009$, and a thickness of t = 0.5 mm. The optimal antenna parameters are presented in Table 1.

**Table 1:** Optimum parameters of the SSPP-based antenna.

Parameter	Value	Parameter	Value
*W*	30 mm	*g*	0.7 mm
*L*	80 mm	*Z*	2.6 mm
*Y*	4.3 mm	*K*	5 mm
*q*	13 mm	*S*	10 mm
*R*	2 mm	*L1*	72 mm
*R1*	4.5 mm		

The proposed antenna consists of three main parts: a CPW feed, an SSPP transmission line, and a tapering area. The CPW feed plays a crucial role in the antenna's functionality as its primary function is to convert the quasi-TEM mode into the SSPP transmission line. Additionally, it serves as a wavelength transformer to achieve impedance matching between the SSPP transmission line and the 50 Ω port. The SSPP transmission line has been used as a transmission line for the excitation of the end-fire antenna due to its advantages such as field confinement, low loss, controllable dispersion, and slow wave. The half-circular tapering region at the end of the antenna is used to convert the TM waves propagated in the SSPP transmission line into free-space propagation waves, and by using a tapering area at the end of the antenna, a wide impedance bandwidth is achieved.

In summary, the wideband characteristic of the proposed antenna design is realized through a combination of key design elements and careful optimization. The antenna design utilizes a CPW feed, an SSPP transmission line, and a tapering area to achieve its wideband operation. The SSPP transmission line provides field confinement, low loss, controllable dispersion, and slow wave, which contribute to the wideband operation of the antenna. The geometry of the unit cell, the length of the tapering half-circles, and the total length of the transmission line cells have been carefully optimized to minimize reflections and impedance variations while maximizing antenna performance. These optimizations are critical for achieving the wideband characteristic of the antenna. Furthermore, the tapering area at the end of the antenna is used to convert the TM waves propagated in the SSPP transmission line into free-space propagation waves, enabling a wide impedance bandwidth.

**Figure 2:**
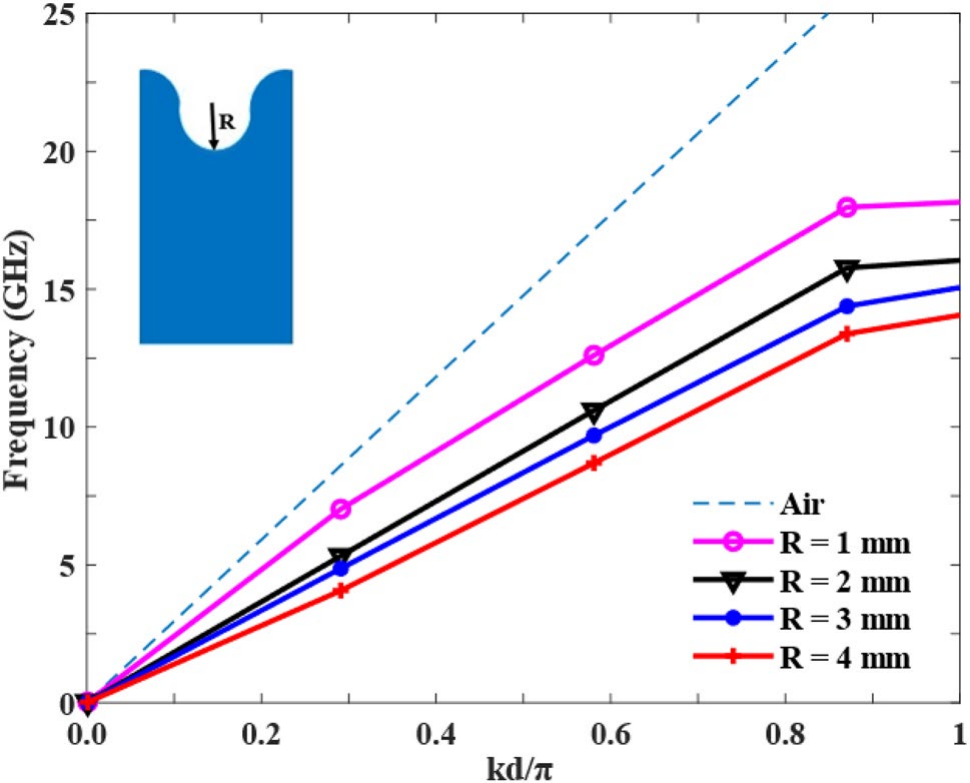
Dispersion curve of the unit cell.

**Figure 3:**
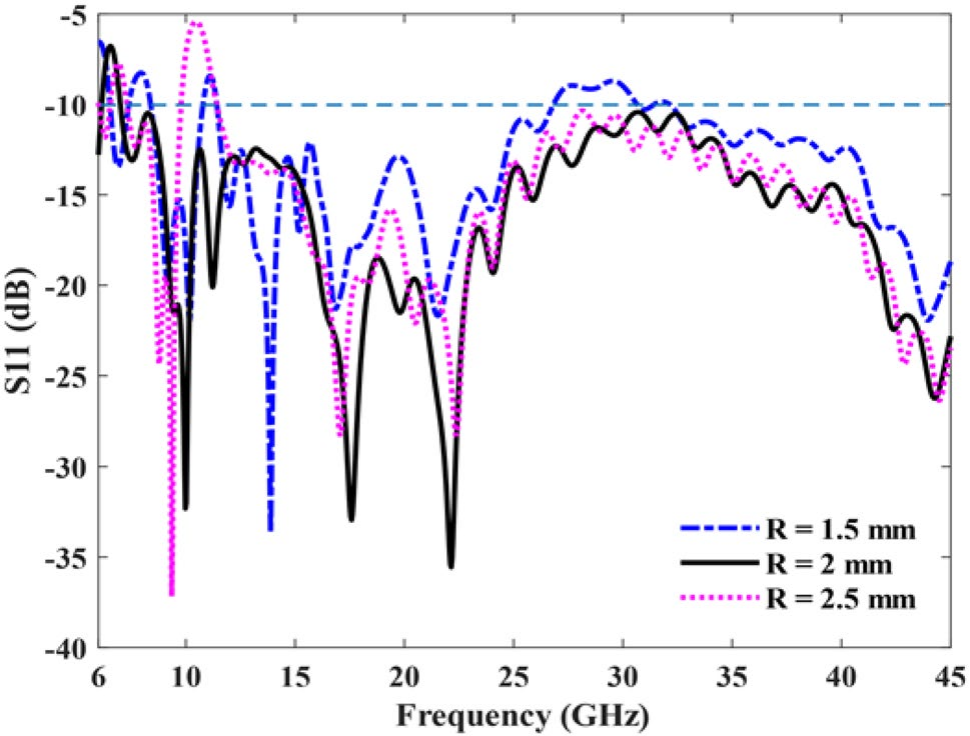
Antenna S11 for different values of R.

The geometry of the half-circular unit cell is defined by two parameters: the radius (R) and the total height (S) of the structure. The proposed antenna operates based on the Hanson-Woodyard^24^ condition, which indicates that not only the terminal part but also the initial and body parts contribute to enhancing the radiation. Also, according to the Hanson-Woodyard condition, the optimal guided phase constant should be slightly larger than that in air. Therefore, the spray curve should remain a short distance below the air line. Figure 2 shows the dispersion curve of the unit cell for varying groove depths, and as illustrated in the figure, the dispersion curve of the SSPP transmission line exhibits a deviation from the light line. This indicates the presence of a slow-wave phenomenon in this structure. As the value of R increases, the field confinement around the SSPP transmission line becomes stronger, resulting in enhanced radiation, improved impedance bandwidth, and increased antenna gain. Moreover, due to the increase in R value from a certain point onward, the spray curve moves away from the air line. Consequently, this results in diminished phase matching between the structure and free space for radiation. Likewise, the value of R significantly influences the impedance matching performance, where Fig. 3 shows the effect of the depth of the groove on the impedance matching. Therefore, there is an optimal groove depth (R = 2 mm) in the desired frequency range.

**Figure 4:**
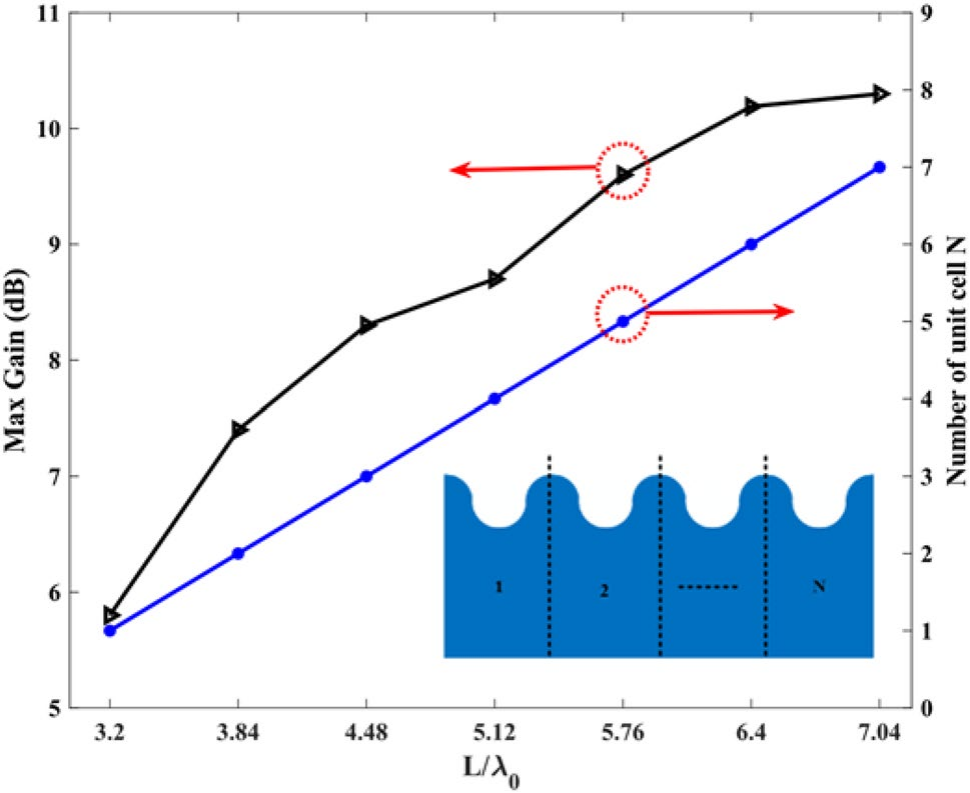
Total length versus maximum gain and number of unit cells (N), where λ0 is the wavelength at 24 GHz.

**Figure 5:**
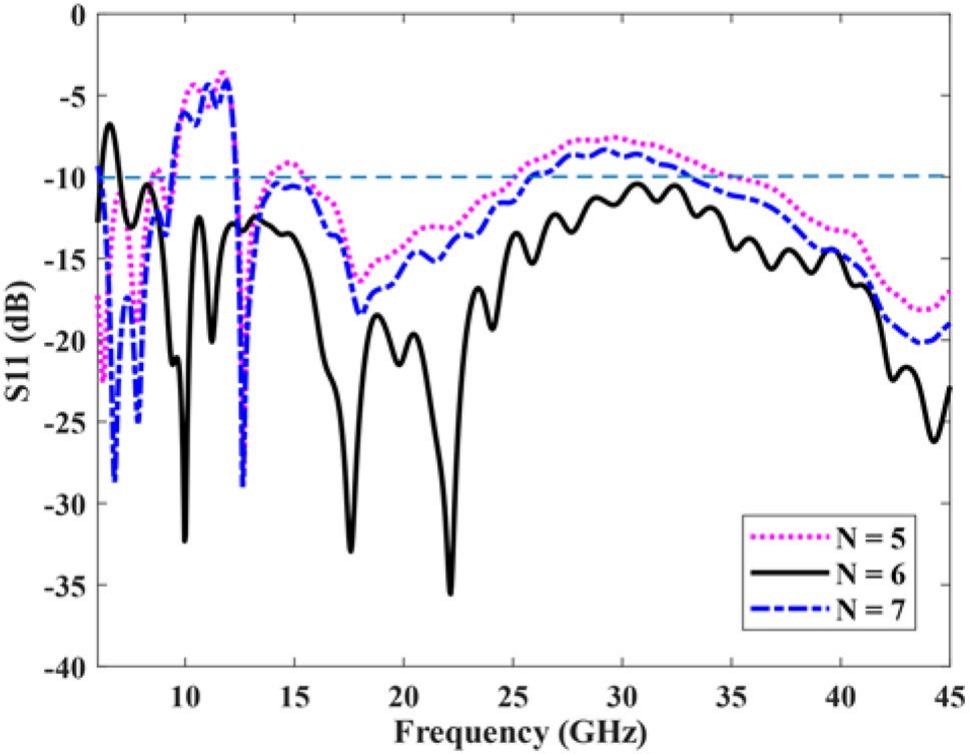
S11 per number of cells.

The height of the tapering half-circles gradually changes with a step of 0.5 mm, within the range of 1.5 mm to 0.5 mm. The value of S also plays a crucial role in impedance matching, and its optimal value is S = 10 mm. The SSPP transmission line, the tapered area at the end of the antenna, and the value of S were optimized to minimize reflections and impedance variations. Furthermore, the length of L1 has been carefully selected to achieve two goals: reducing reflections and improving impedance matching without increasing the size of the antenna. Hence, the optimal value for L1 is determined to be 72 mm. It is important to mention that to finalize the remaining dimensions of the proposed antenna, all the parameters have been determined based on thoroughly examination and extensive simulations. To optimize the antenna’s response, it is crucial to take into account the structural size of each component and its impact on the overall performance of the antenna. Therefore, it is essential to optimize the number of transmission line cells to achieve the best results. Figure 4 illustrates the variation in maximum antenna gain with respect to the total length, represented by the number of SSPP transmission line cells. The gain shows an increase from 5.8 dB to 10.19 dB as the antenna length increases. Notably, when the length ratio L/λ0 exceeds 6.4, there is a slight increase in the maximum gain. Based on this observation, a transmission line consisting of six unit cells was selected to achieve the highest gain while maintaining a minimal antenna size. Consequently, the total length of the line has been determined as L = 80 mm. Also, Fig. 5 shows the reflection coefficients of the antenna per unit cell number. As can be seen, the reflection coefficients of the antenna per 6 cells have better impedance matching than others.

**Figure 6:**
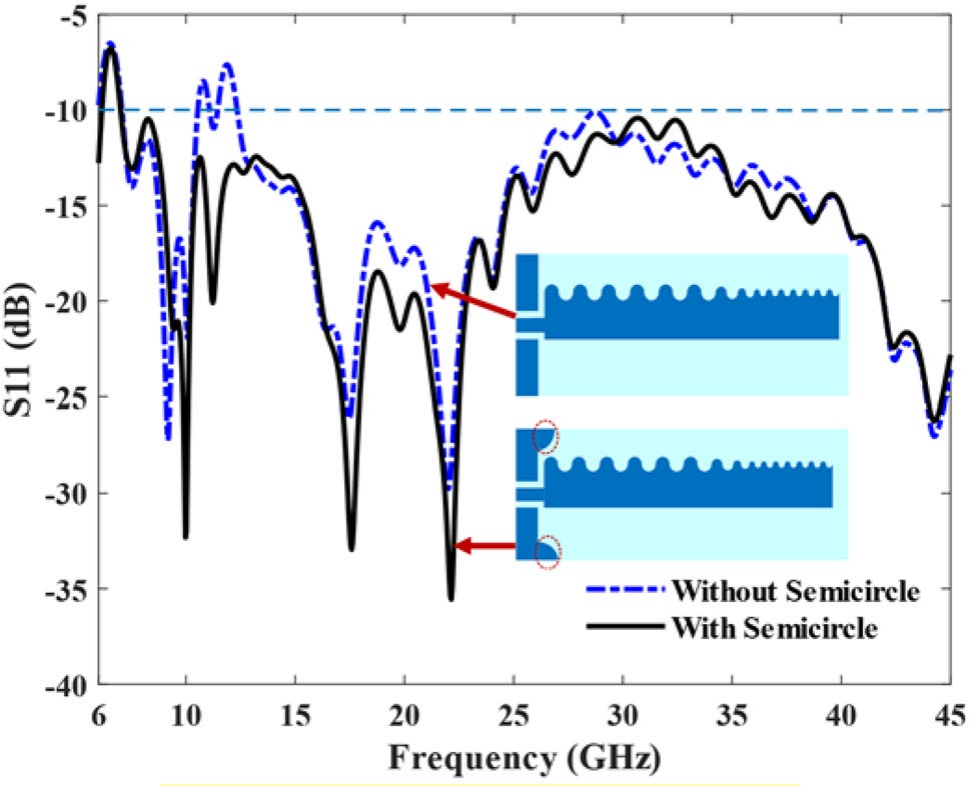
Antenna S11 with and without a half-circle.

Figure 6 illustrates the reflection coefficients of the antenna with and without the half-circle introduced in front of the antenna's ground plane. The half-circle acts as a reflector, causing transmitted energy to reflect back and this results in improved reflection coefficients, particularly at lower frequencies. The reflection coefficients of the antenna without the half-circle are observed within the frequency range of 11.1 to 45 GHz, whereas the antenna with the half-circle exhibits better impedance matching in the frequency range of 7 to 45 GHz.

**Figure 7:**
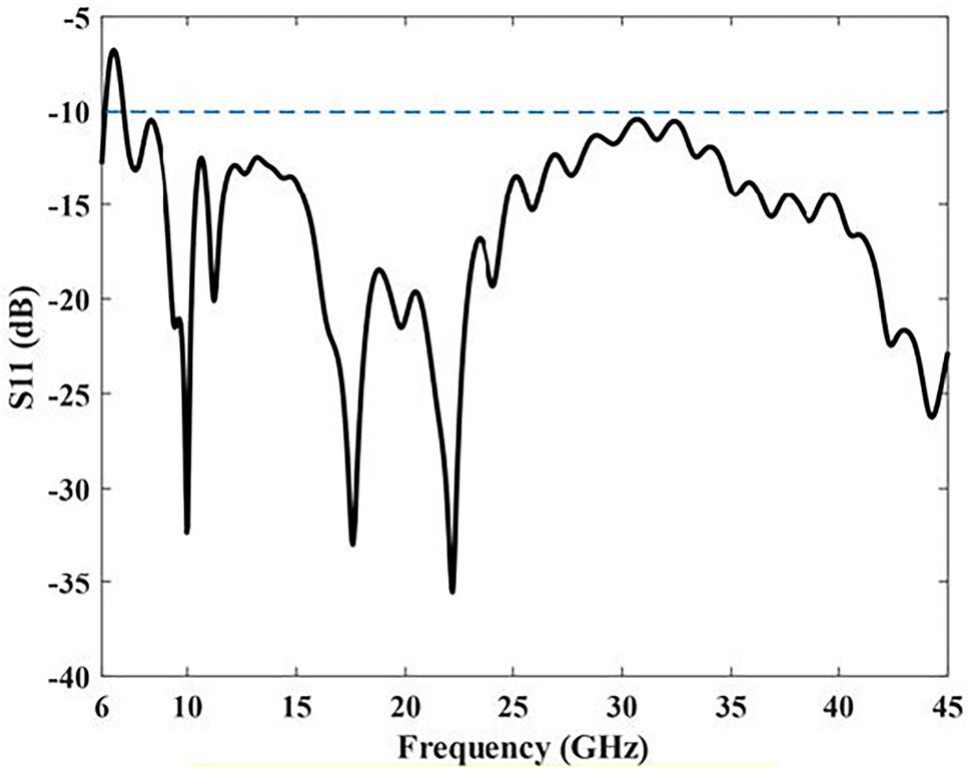
Simulated S11 of the proposed antenna.

The proposed design has been implemented and simulated using CST software, utilizing a Rogers RT5880 substrate. Figure 7 displays the reflection coefficients of the proposed antenna, revealing that the reflection coefficients remain below -10 dB in the frequency range of 7 to 45 GHz, indicating good impedance matching within this range.

**Figure 8:**
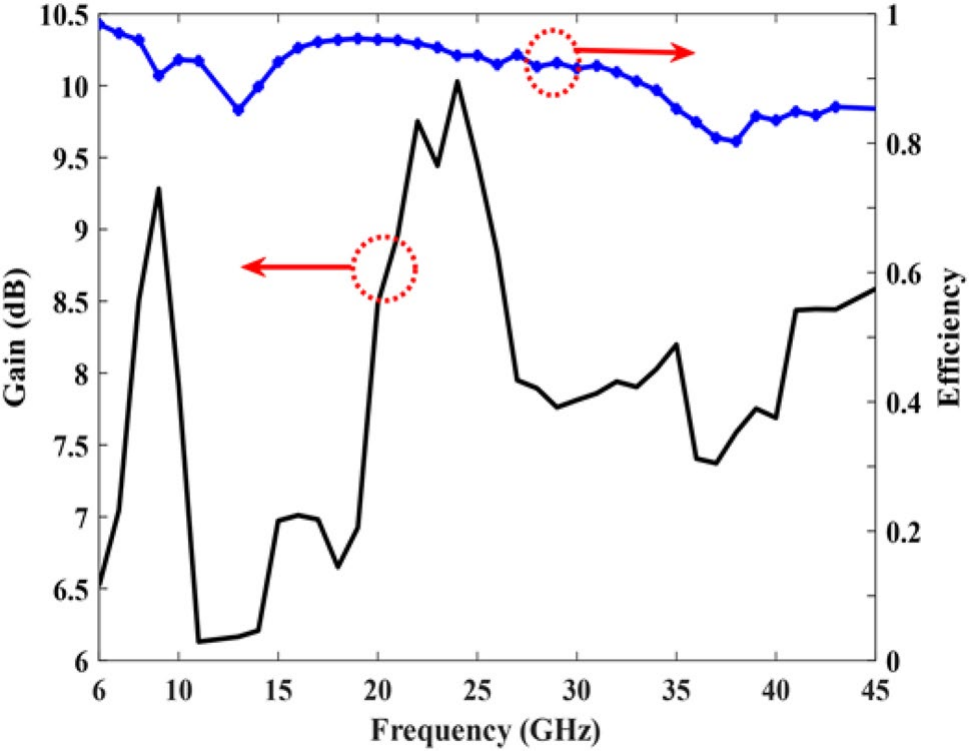
Simulated gain and efficiency of the proposed antenna..

Figure 8 illustrates the gain and efficiency of the simulated antenna across the frequency range of 7 to 45 GHz where the total gain exhibits variations from 6.13 to 10.19 dB. The simulation results clearly demonstrate that the proposed antenna achieves high efficiency, averaging 90% across a wide operating band from 7 to 45 GHz. In Fig. 8, we evaluated the gain of the structure. However, upon factoring in the negative impact of reflection loss, we discovered that realized gain was 0.25dB lower than the calculated gain.

## Fabrication and measurement

**Figure 9:**
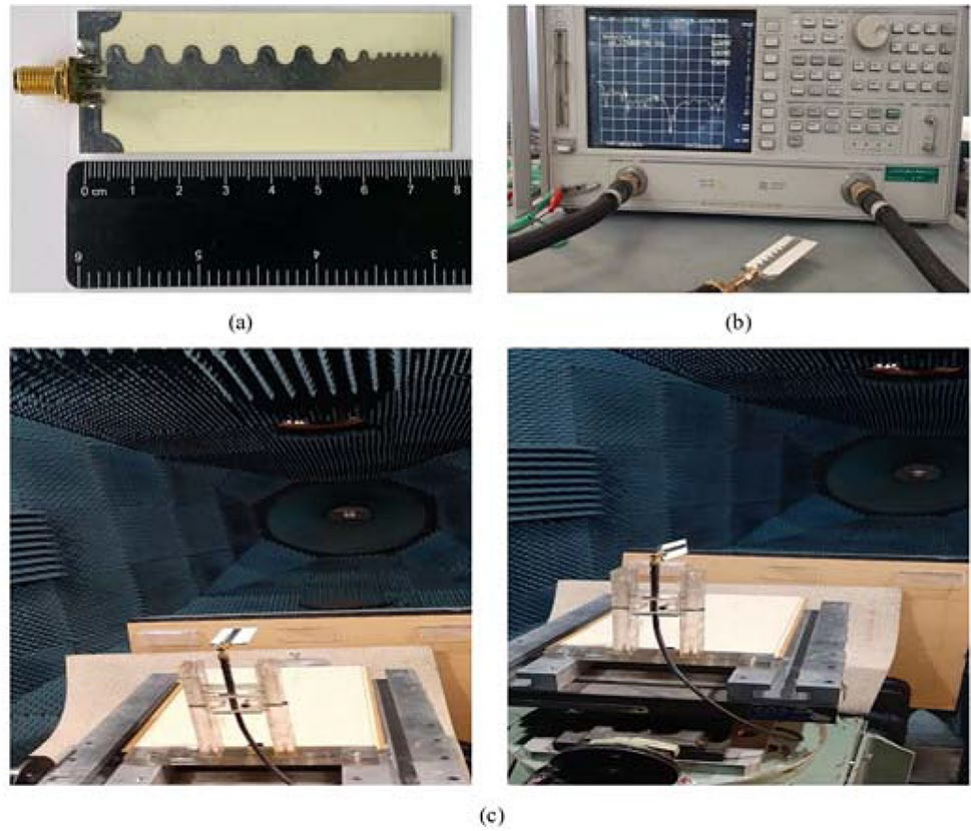
Prototype of the built antenna. (a) General view of the built antenna. (b) View of the antenna connected to the network analyzer. (c) View of the antenna in the antenna room.

We have designed and simulated the proposed scheme using Rogers Ro5880 substrate and have achieved an acceptable bandwidth. However, in the manufacturing phase, due to the limitation in the supply of Rogers Ro5880 substrate as well as the limitation in measurement equipment, we had to do a redesign on the Rogers Ro4003c substrate with εr = 3.55, tan δ = 0.0027, and thickness of 0.5 mm, as shown in Fig. 9. Measurements on this substrate have been possible up to the maximum frequency of 22 GHz.

**Figure 10:**
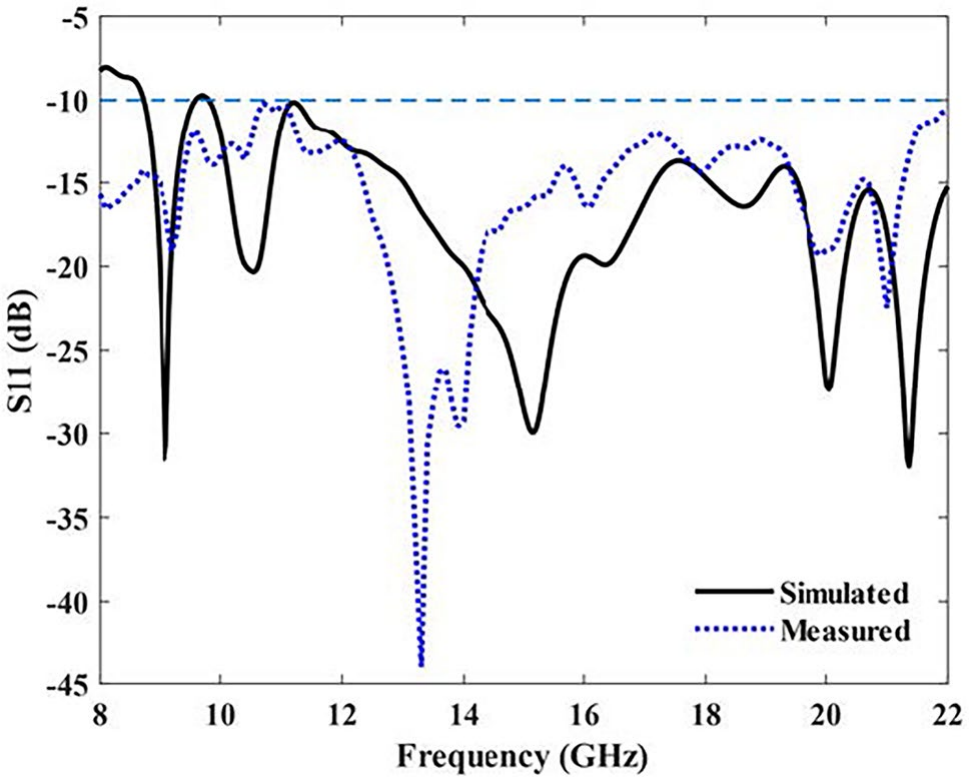
Simulated and measured S11 of the SSPP-based antenna.

To accommodate the limited frequency range of the spectrum analyzer, the reflection coefficients of the proposed antenna were plotted within the frequency range of 8–22 GHz. Figure 10 shows the reflection coefficient results obtained from the simulation and measurement, which exhibited consistency with each other and confirmed the accuracy of the antenna design. The frequency deviation observed can be attributed to manufacturing errors. Notably, the reflection coefficients remained below −10 dB within the range of 8.7 to 22 GHz, indicating good impedance matching.

**Figure 11:**
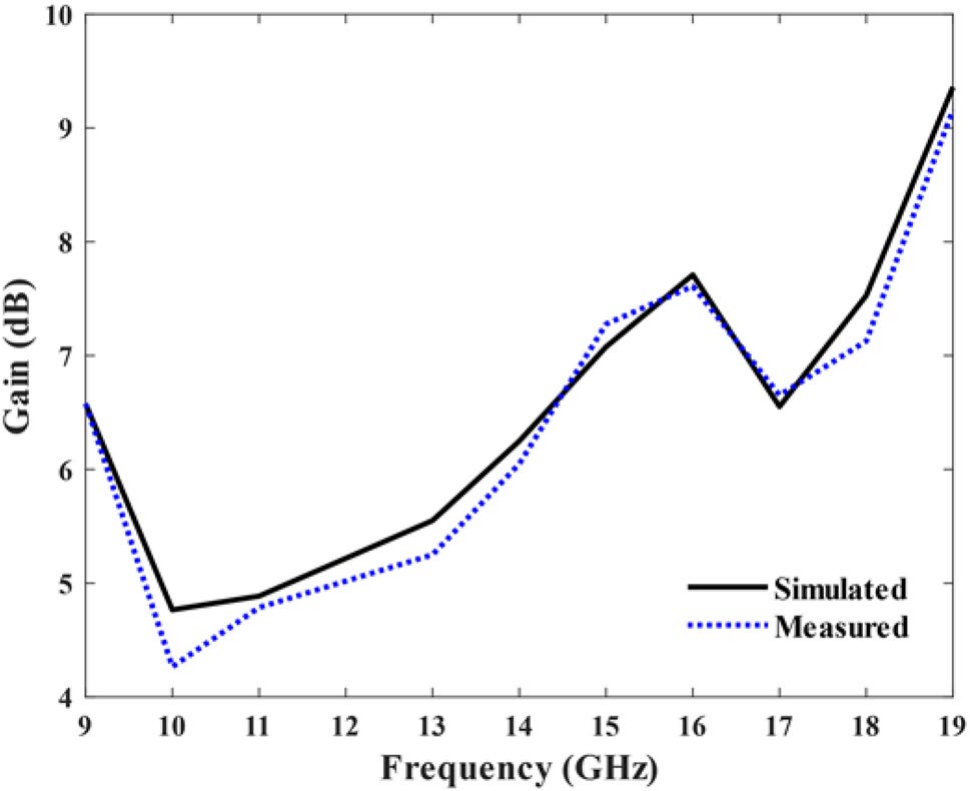
Simulated and measured gain of the SSPP-based antenna.

**Figure 12:**
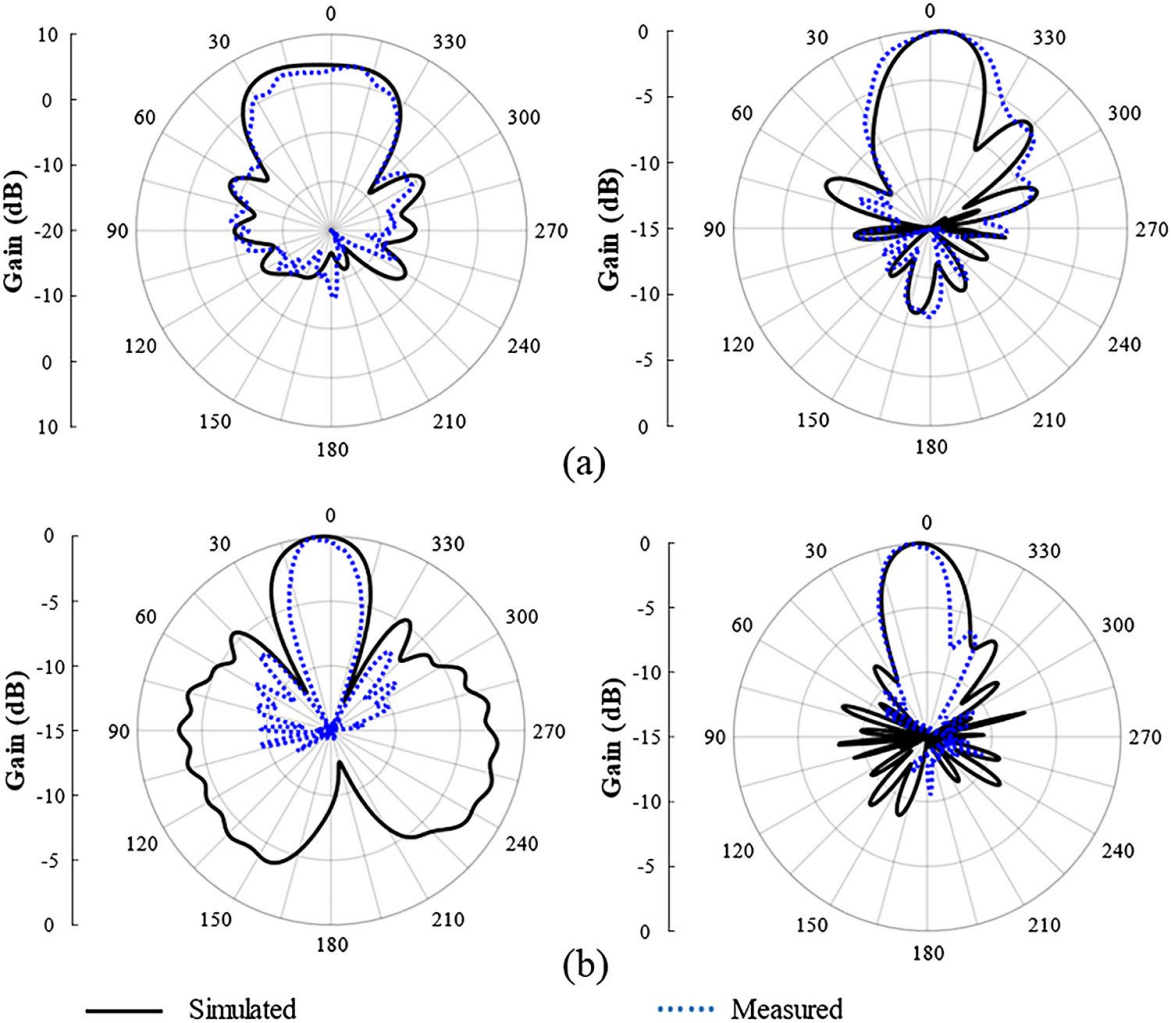
Simulation and measurement of the radiation fields of plane E and plane H. [(right) page E; (left) page H]. (**a**) 6 GHz. (**b**) 6.8 GHz.

Figure 11 illustrates the simulated and measured antenna gain within the frequency range of 9 to 19 GHz. The total gain varies from 4.7 to 9.1 dB, and the results obtained from both simulation and measurements align with each other. Moreover, the simulated and measured normalized electric field (E) and magnetic field (H) radiation patterns at frequencies of 6 and 6.8 GHz are illustrated in Fig. 12. Table 2 depicts the comparison of our proposed antenna with other works in the literature.

**Table 2:** A comparative analysis with other sources.

Ref	Bandwidth (%)	Efficiency (%)	Gain (dB)	Frequency GHz)	Size (mm)
^16^	*8.5*	*9.32*	*7*	*6*	*113* $\times$ *32*
^18^	*31*	*95*	*9.16*	*7*	*133* $\times$ *32*
^19^	*70.3*	*90*	*8.6*	*3.7*	*110* $\times$ *70*
^20^	*67*	*54*	*7.9*	*6*	*116* $\times 40$
^22^	*66*	*96*	*9.2*	*9*	*107* $\times$ *30*
^25^	*73*	*87.5*	*12.4*	*9.5*	*75.5* $\times$ *20*
^26^	*11*	*95*	*12.1*	*16.6*	*167* $\times$ *80*
This Work	*146*	*90*	*10.19*	*26*	*80* $\times$ *30*

## Conclusion

This paper has presented the design, simulation, and fabrication of a compact antenna based on an SSPP structure, where an SSPP waveguide has been utilized for the transmission line, enabling a wide bandwidth through field confinement, slow waves, and low losses. The proposed antenna was designed on a Rogers Ro5880 substrate, yielding simulation results of a 146% bandwidth, 10.19 dB maximum gain, and 90% efficiency. However, due to manufacturing limitations and the high cost associated with the Rogers Ro5880 substrate, our proposed antenna was instead fabricated and measured on a Rogers Ro4003c substrate. The results obtained from simulation and measurement on the Rogers Ro4003c substrate have demonstrated an 87% bandwidth within the frequency range of 8.5 to 22 GHz, with a maximum gain of 9.1 dB. The results obtained from simulation and measurement have also shown good agreement. Moreover, due to the unique characteristics of the SSPP transmission line, the proposed antenna demonstrates a wide bandwidth, high gain, high efficiency, and a compact size. These features position the antenna as a good candidate for wireless applications.

## Data Availability

The datasets generated and analyzed during the current study are available from the corresponding author on reasonable request.
